# Jmjd3 Mediates Neuropathic Pain by Inducing Macrophage Infiltration and Activation in Lumbar Spinal Stenosis Animal Model

**DOI:** 10.3390/ijms222413426

**Published:** 2021-12-14

**Authors:** Jeeyoun Lee, Haeyoung Choi, Chansol Park, Sangryong Jeon, Taeyoung Yune

**Affiliations:** 1 Age-Related and Brain Diseases Research Center, Kyung Hee University, Seoul 02447, Korea; jeeyoun@khu.ac.kr (J.L.); neuron@khu.ac.kr (H.C.); chansol1028@khu.ac.kr (C.P.); 2 Department of Neurological Surgery, Asan Medical Center, University of Ulsan College of Medicine, 88, Olympic-ro 43-gil, Songpa-gu, Seoul 05505, Korea; srjeon@amc.seoul.kr; 3 Department Biochemistry and Molecular Biology, School of Medicine, Kyung Hee University, Seoul 02447, Korea

**Keywords:** lumbar spinal stenosis, cauda equina, neuropathic pain, inflammation, Jmjd3, blood-nerve barrier

## Abstract

Lumbar spinal stenosis (LSS) is a major cause of chronic neuropathic back and/or leg pain. Recently, we demonstrated that a significant number of macrophages infiltrated into the cauda equina after compression injury, causing neuroinflammation, and consequently mediating neuropathic pain development and/or maintenance. However, the molecular mechanisms underlying macrophage infiltration and activation have not been elucidated. Here, we demonstrated the critical role of histone H3K27 demethylase Jmjd3 in blood-nerve barrier dysfunction following macrophage infiltration and activation in LSS rats. The LSS rat model was induced by cauda equina compression using a silicone block within the epidural spaces of the L5-L6 vertebrae with neuropathic pain developing 4 weeks after compression. We found that Jmjd3 was induced in the blood vessels and infiltrated macrophages in a rat model of neuropathic pain. The blood-nerve barrier permeability in the cauda equina was increased after compression and significantly attenuated by the Jmjd3 demethylase inhibitor, GSK-J4. GSK-J4 also inhibited the expression and activation of MMP-2 and MMP-9 and significantly alleviated the loss of tight junction proteins and macrophage infiltration. Furthermore, the activation of a macrophage cell line, RAW 264.7, by LPS was significantly alleviated by GSK-J4. Finally, GSK-J4 and a potential Jmjd3 inhibitor, gallic acid, significantly inhibited mechanical allodynia in LSS rats. Thus, our findings suggest that Jmjd3 mediates neuropathic pain development and maintenance by inducing macrophage infiltration and activation after cauda equina compression and thus may serve as a potential therapeutic target for LSS-induced neuropathic pain.

## 1. Introduction

Lumbar spinal stenosis (LSS) is a degenerative condition in which the vertebral canal becomes narrow due to the common occurrence of a slipped disk, lumbar spondylolisthesis, ligamentous thickening, and spinal degeneration with aging [1]. The patients often experience pain in the legs and back, intermittent claudication, walking disability, numbness, and weakness of the legs. As a result, LSS patients become unable to perform daily activities and face psychological difficulties, such as insomnia, depression, anxiety, and social isolation [1,2]. In particular, the compression of cauda equina fibers in chronic LSS patients induces hypersensitivity and sensitization of the central nervous system (CNS) and peripheral nervous system (PNS), which causes generalized and severely debilitating neuropathic pain in millions of affected people worldwide. Several medications, such as antidepressants, anti-seizure drugs, and opioids, are used to reduce LSS-induced neuropathic pain [3,4,5]. However, LSS-induced neuropathic pain cannot be adequately alleviated by currently available medications. Thus, additional therapeutic drug development should be considered for patients with neuropathic pain.

In general, it has been known that the up-regulation of neuroinflammatory reactions plays a key role in acute and chronic pain syndromes. After a spinal cord or nerve injury, immune cells migrate to the injury site, producing pro-inflammatory cytokines, mediating inflammatory reactions. This inflammatory response eventually reduces the firing thresholds of A-σ and C-fiber nociceptors, leading to neuropathic pain [6,7,8,9]. In the LSS animal model, it was observed that many immune cells, such as macrophages, infiltrated into both the spinal cord and cauda equina [10,11]. Recently, we reported that macrophages infiltrated into the cauda equina one month after compression of the cauda equina in the LSS model. Furthermore, COX-2 expression was increased in the infiltrated macrophages, and the inflammatory response and chronic neuropathic pain were significantly alleviated by treating the COX-2 inhibitor, celecoxib [12]. Our findings suggest that immune cell infiltration into the cauda equina may be associated with the development and maintenance of neuropathic pain caused by LSS. 

The blood–nerve barrier is a dynamic and competent interface between the endoneurial microenvironment and the surrounding extracellular space or blood. The blood-nerve barrier as a barrier system has almost the same properties as the blood-brain barrier/blood-spinal cord barrier [13,14]. In LSS patients, it has been known that increasing mechanical compression promotes the disturbance of CSF flow, circulatory disturbance starting from the venous congestion, and the breakdown of the blood-nerve barrier in the cauda equina [15]. While little is known about the mechanism of damage to the blood-nerve barrier in LSS patients, it is known that matrix metalloproteinase (MMP)-2 and MMP-9 are produced and involved in the blood-nerve barrier breakdown, immune cell recruitment, and pain development after peripheral nerve injury [16,17,18]. In addition, our previous report showed that histone H3K27 demethylase Jmjd3 plays a critical role in regulating blood-spinal cord barrier integrity by activating the MMP-2, -3, and -9 after spinal cord injury [19]. We recently reported that gallic acid protected the integrity of vascular endothelial cells after oxygen-glucose deprivation (OGD) through the inhibition of Jmjd3 activity and significantly inhibited blood-spinal cord barrier damage after spinal cord injury [20]. Based on this evidence, we questioned whether Jmjd3 might induce blood-nerve barrier damage and promote macrophage infiltration into the cauda equina, thereby mediating neuropathic pain in the LSS animal model.

Here, we examined whether Jmjd3 plays an important role in blood-nerve barrier integrity in the cauda equina after compression injury and mediates LSS-induced neuropathic pain. Thus, the present study was designed to examine whether: (1) the expression and activity of Jmjd3 are increased in the LSS animal model; (2) Jmjd3 is involved in blood-nerve barrier damage in the injured cauda equina; and (3) Jmjd3 mediates neuropathic pain development and maintenance through modulating macrophage infiltration and neuroinflammation.

## 2. Results

### 2.1. Jmjd3 Expression and Activity Is Increased after Cauda Equine Compression in an LSS Model

**Figure 1:**
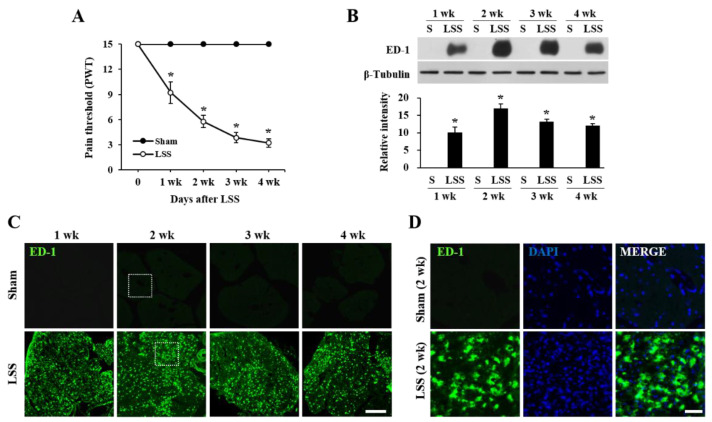
Neuropathic pain development is correlated to the temporal pattern of macrophage infiltration in the cauda equina after compression. (**A**) Time course of change in pain threshold of the hind limb. Mechanical allodynia was assessed by the paw withdrawal threshold (PWT) in response to von Frey filaments (n = 10/group). The data are presented as the mean ± SEM. * *p* < 0.05 vs. Sham. (**B**) Western blots for ED-1 and quantitative analysis of Western blots at indicated time points after the cauda equina compression. The data are presented as the mean ± SD (n = 3). * *p* < 0.05 vs. Sham (S). (**C**) Representative immunofluorescence staining for ED-1 positive macrophage in sham and injured cauda equina at indicated time points after compression. Scale bars, 100 μm. (**D**) Higher magnification images of the boxed area in Figure 1C with DAPI (blue). Scale bars, 50 μm.

To determine the spatiotemporal dynamics of macrophage infiltration after compression injury, we first examined the responses to innoxious, mechanical stimuli using a von Frey filament. The paw withdrawal threshold (PWT) gradually decreased from 1 week after compression injury, and significant tactile allodynia developed through 4 weeks (PWT, 3.2 ± 0.3) (Figure 1A, LSS). However, no change in PWT was observed in uninjured rats (Figure 1A, Sham). Next, to examine the temporal pattern of macrophage infiltration in the cauda equina, we assessed a Western blot and immunohistochemistry for ED-1, a macrophage marker, at 1, 2, 3, and 4 weeks after the compression injury. Western blot analysis revealed that the level of ED-1 was markedly increased at week 1 and peaked at 2 weeks after injury, with an expression level maintained until 4 weeks, while the pain threshold started to decrease at 1 week and further decreased until 4 weeks (Figure 1B). Immunohistochemistry also revealed that ED-1 positive macrophages were observed throughout the compressed cauda equina (Figure 1C,D).

**Figure 2:**
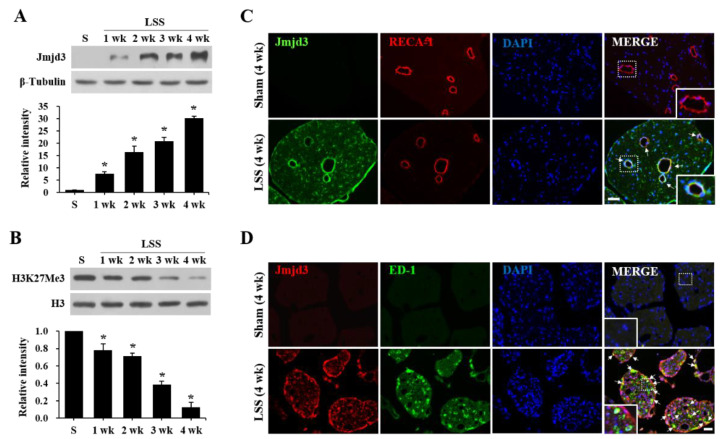
Jmjd3 expression and activation are increased in the cauda equina after compression injury in an LSS model. (**A**,**B**) Western blot and quantitative analysis for Jmjd3 (**A**) and H3K27Me3 (**B**) in the cauda equina at an indicated time point after the compression injury. The data are presented as the mean ± SD (n = 3). * *p* < 0.05 vs. Sham (4 weeks). (**C**) Double immunofluorescence staining for Jmjd3 and RECA-1 (endothelial cell marker) in Sham (4 weeks) and injured cauda equina (4 weeks after compression). Arrows indicate double-positive cells. The right, bottom panels show a higher magnification of the boxed area. Scale bar, 50 μm. (**D**) Double immunofluorescence staining for Jmjd3 and ED-1 (macrophage marker) in Sham (4 weeks) and injured cauda equina (4 weeks after compression). Arrows indicated double-positive cells. The left, bottom panels show a higher magnification of the boxed area. Scale bar, 50 μm.

To determine the expression and activation profile of Jmjd3 in the cauda equina after injury, we performed a Western blot for Jmjd3. Jmjd3 was not detectable in the sham control. After the compression injury, Jmjd3 significantly increased from 1 week and further increased until 4 weeks (Figure 2A). Since it is known that Jmjd3 catalyzes the demethylation of H3K27Me3 and regulates gene transcription, we examined the change of H3K27Me3 level by Western blot. As shown in Figure 2B, the level of H3K27Me3 was also significantly decreased in a time-dependent manner, indicating an increase in Jmjd3 activity. Immunohistochemistry showed that Jmjd3 immunoreactivity was not detected in the sham cauda equina (Figure 2C, Sham). However, strong Jmjd3 immunoreactivity was observed in blood vessels and round-shaped cells in the injured cauda equina at 4 weeks after compression. Double immunofluorescence labeling revealed that Jmjd3 immunoreactivity was observed in blood vessels (RECA1-positive) and macrophages (ED-1-positive) (Figure 2C,D). These results suggest that Jmjd3 expression is up-regulated in the endothelial cells of blood vessels and infiltrated macrophages in the cauda equina after compression.

### 2.2. Jmjd3 Regulates Blood-Nerve Barrier Permeability after Cauda Equine Compression in an LSS Model

**Figure 3:**
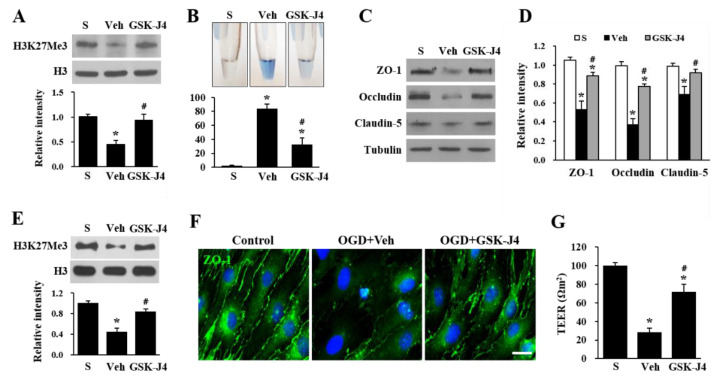
Jmjd3 regulates blood-nerve barrier permeability after cauda equine compression. At 30 d after the compression injury, GSK-J4 (20 µg) or vehicle (Veh) was intrathecally injected, and the cauda equina was prepared 30 min after drug injection. (**A**) Western blot and quantitative analysis for H3K27Me3. The data are presented as the mean ± SD (n = 3). (**B**) Representative cauda equina homogenates in e-tube (upper panel) and quantification (bottom panel) of the amount of Evans blue dye extravasation 30 d after compression. Data represent as mean ± SEM (n = 5). * *p* < 0.05 vs. Sham; # *p* < 0.05 vs. Vehicle. (**C**) Western blots for ZO-1, occludin, and claudin-5 in the cauda equina. (**D**) Quantitative analysis of Western blots. The data are presented as the mean ± SD (n = 3). * *p* < 0.05 vs. Sham; # *p* < 0.05 vs. Vehicle. (**E**–**G**) bEnd.3 cells were pretreated with vehicle or GSK-J4 (4 μM) for 30 min before oxygen-glucose deprivation (OGD) for 6 h. (**E**) Western blot and quantitative analysis for H3K27Me3 at 1 h reoxygenation after OGD. The data are presented as the mean ± SD (n = 3). * *p* < 0.05 vs. Sham; # *p* < 0.05 vs. Vehicle. (**F**) Immunocytochemistry for ZO-1 from fixed bEnd.3 cells at 6 h reoxygenation after OGD. Scale bar, 30 μm. (**G**) After being treated with control or GSK-J4, bEnd.3 cells were subjected to OGD/reperfusion injury, and TEER was measured at reperfusion 1 h after OGD, as described in the Methods section. The data are presented as the mean ± SD (n = 3). * *p* < 0.05 vs. Sham; # *p* < 0.05 vs. Vehicle.

Data show that Jmjd3 expression increased in blood vessels in the cauda equina which led us to postulate that Jmjd3 would induce blood-nerve barrier damage, followed by increased permeability after a compression injury. Therefore, the effect of GSK-J4 (an H3K27 demethylase inhibitor) on Evans blue dye extravasation was examined to determine the role of Jmjd3 in blood-nerve barrier dysfunction. GSK-J4 was injected intrathecally 30 days (d) after caudal compression and its effect was evaluated after 30 min. As shown in Figure 3A, the level of H3K27Me3 decreased in the compression injured cauda equina, while GSK-J4 treatment significantly attenuated the decrease of H3K27Me3 level as compared with the vehicle control, without affecting the total level of histone H3. When we determined blood-nerve barrier permeability through an Evans blue assay, the amount of Evans blue dye extravasation was markedly increased at 30 d after the compression injury, which implies a blood-nerve barrier leakage. Furthermore, the level of Evans blue dye extravasation was significantly diminished in GSK-J4-injected rats as compared with the vehicle-injected rats. However, any signal of Evans blue was not detected in the sham control (Figure 3A,B). To confirm the effect of Jmjd3 on blood-nerve barrier permeability, we investigated the level of tight junction proteins in endothelial cells, which are important factors regulating blood-nerve barrier permeability. Western blots showed that the levels of ZO-1, occludin, and claudin-5 were markedly decreased in the cauda equina 30 d after injury. However, the levels of ZO-1, occludin, and claudin-5 were significantly higher in the GSK-J4 injected rats than in vehicle-injected rats (Figure 3C,D), suggesting that Jmjd3 regulates the loss of tight junction proteins of endothelial cells in the injured cauda equina. To determine whether Jmjd3 directly regulates endothelial cell hyperpermeability, we applied OGD and reoxygenation to bEnd.3 cells, a mouse brain endothelial cell line. As shown in Figure 3E, Western blot showed that the level of H3K27Me3 markedly decreased at 1 h of reoxygenation after OGD, whereas the decrease of H3K27Me3 level was significantly inhibited by GSK-J4 (Figure 3E). The intensity of ZO-1 expression by immunocytochemistry was also decreased by OGD/reoxygenation as compared to control cells, and the loss of ZO-1 intensity was alleviated by GSK-J4 treatment (Figure 3F). Next, we investigated the change in tight junction integrity by measuring TEER. Similar to the results of ZO-1 expression, TEER was markedly decreased by OGD/reperfusion injury, but GSK-J4 treatment significantly alleviated the decrease of TEER compared with vehicle-treated cells (Figure 3G). These results imply that Jmjd3 mediates the decrease of tight junction protein level and endothelial permeability after injury.

### 2.3. Jmjd3 Inhibitor Reduces Macrophage Infiltration and Inflammation in Injured Cauda Equina of LSS Rats

**Figure 4:**
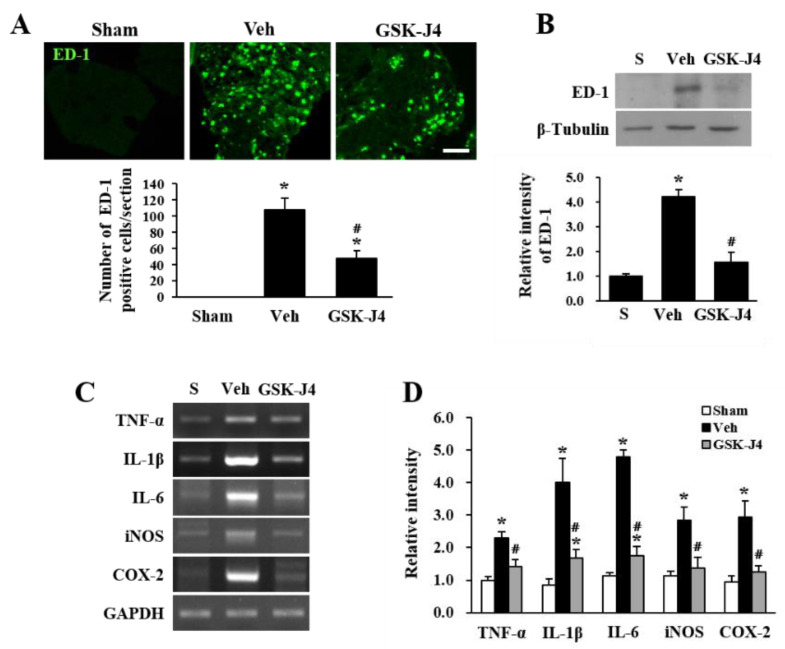
The Jmjd3 inhibitor reduced macrophage infiltration and inflammation in the injured cauda equina of LSS rats. (**A**) Immunofluorescence staining for ED-1 in Sham, vehicle- or GSK-J4-injected rat at 30 min after treatment (upper panel). Quantitative analysis of the number of ED-1 positive cells per section. The data are presented as the mean ± SD (n = 3). * *p* < 0.05 vs. Sham; # *p* < 0.05 vs. Vehicle. Scale bar, 50 μm. (**B**) Western blot and quantitative analysis for ED-1 30 min after vehicle or GSK-J4 treatment. The data are expressed as the mean ± SD (n = 3). * *p* < 0.05 vs. Sham; # *p* < 0.05 vs. Vehicle. (**C**) RT-PCR for TNF-α, IL-1β, IL-6, iNOS, and COX-2 in the cauda equina 30 min after vehicle or GSK-J4 treatment. (**D**) Quantitative analysis of RT-PCR. The data are expressed as the mean ± SD (n = 3). * *p* < 0.05 vs. Sham; # *p* < 0.05 vs. Vehicle.

Since we showed that GSK-J4 treatment inhibits blood-nerve barrier hyperpermeability in the cauda equina in LSS rats (Figure 3), we next examined whether the inhibition of Jmjd3 would also attenuate macrophage infiltration. We performed immunohistochemistry using an ED-1 antibody for macrophage staining. As shown in Figure 4A, the number of ED-1 positive macrophages was significantly decreased in the GSK-J4 injected cauda equina at 30 d after injury compared to the vehicle-injected cauda equina (Vehicle, 108 ± 13 cells; GSK-J4, 47 ± 9 cells; *p* ≤ 0.05) (Figure 4A). We further confirmed that the level of ED-1 expression was significantly decreased in the GSK-J4 treated cauda equina as compared to the vehicle-injected cauda equina after LSS by Western blot analysis (Figure 4B). Because the infiltration of macrophages in LSS rats mediates inflammatory responses [12], we also examined the expression of inflammatory mediators by RT-PCR. The gene expression levels of TNF-α, IL-1β, IL-6, iNOS, and COX-2 were up-regulated in the cauda equina after compression injury, but GSK-J4 treatment significantly reduced their expression levels compared to vehicle-injected cauda equina (Figure 4C,D).

### 2.4. Jmjd3 Regulates the Expression and Activity of MMP-2/9 in Cauda Equina after Compression Injury

**Figure 5:**
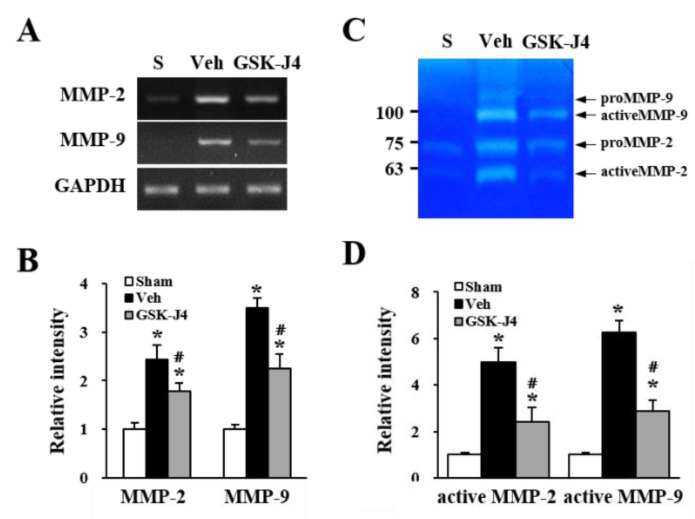
Jmjd3 regulates the expression and activity of MMP-2/9 in the cauda equina after a compression injury. At 30 d after compression injury, GSK-J4 or vehicle-treated cauda equina were prepared 30 min after drug injection and RT-PCR and gelatin zymography were assessed. (**A**) RT-PCR for MMP-2 and MMP-9. (**B**) Quantitative analysis of RT-PCR (n = 3). (**C**) Gelatin zymography for MMP-2 and MMP-9. (**D**) Quantitative analysis of active MMP-2 and MMP-9 (n = 3). All data are expressed as the mean ± SD. * *p* < 0.05 vs. Sham; # *p* < 0.05 vs. Vehicle.

It has been reported that MMP-2 and MMP-9 can cause the leakage of the blood-brain barrier or blood-spinal cord barrier [21,22], and the mechanism may be mediated by degradation of tight junction proteins in endothelial cells. In addition, our previous report demonstrated that Jmjd3 is required for MMP-2 and MMP-9 gene expression [19]. Therefore, we determined whether GSK-J4 inhibits the expression and activity of MMP-2 and MMP-9 in LSS rats. As shown in Figure 5A, the MMP-2 and MMP-9 mRNA levels were increased 30 d after compression injury compared with the sham control. However, the LSS-induced increase in both MMP-2 and MMP-9 mRNA expression was significantly inhibited by GSK-J4 treatment compared with the vehicle control (Figure 5A,B). Using gelatin zymography, an increase in the activity of MMP-2 and MMP-9 in the cauda equina was also observed 30 d after the compression injury (Figure 5C), and GSK-J4 significantly inhibited these activities compared with the vehicle control (Figure 5C,D).

### 2.5. Jmjd3 Also Regulates Macrophage Activation

**Figure 6:**
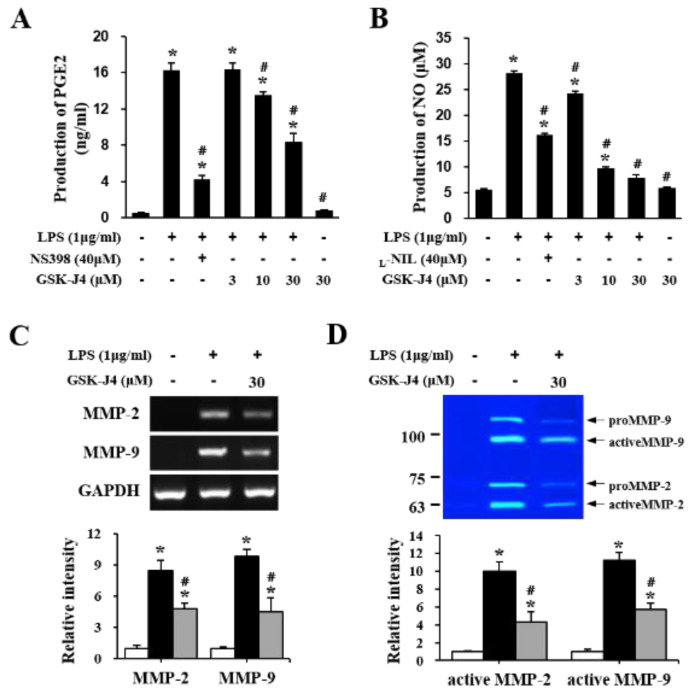
Jmjd3 also regulates macrophage activation in LPS-treated RAW264.7. (**A**) PGE2 production in Raw 264.7 cells by ELISA. Cells were pretreated with different concentrations (3, 10, and 30 μM) of GSK-J4 for 1 h, and then LPS (1 μg/mL) was added and incubated for 24 h. (n = 3). (**B**) NO assay in Raw 264.7 cells at 24 h after LPS treatment. (n = 3). (**C**) RT-PCR and quantitative analysis for MMP-2 and MMP-9 at 12 h after LPS treatment (n = 3). (**D**) Gelatin zymography and quantitative analysis for MMP-2 and MMP-9 24 h after LPS treatment (n = 3). N = 3 means the results in each sample were determined in triplicate (technical replicates) on samples deriving from three independent cultures. All data are presented as the mean ± SD. * *p* < 0.05 vs. Control cells; # *p* < 0.05 vs. LPS only treated cell.

Based on the results that Jmjd3 is expressed not only in blood vessels but also in macrophages in the cauda equina of LSS rats (see Figure 2D), as well as the previous reports showing that Jmjd3 modulates the proinflammatory macrophage response [23,24], we speculated that Jmjd3 might also be involved in the activation of macrophage infiltration in the cauda equina of LSS rats. To determine whether Jmjd3 directly mediates macrophage activation in our in vitro model, we used Raw 264.7, a murine bone marrow-derived macrophage cell line, activated by LPS. As shown in Figure 6, LPS stimulation induced prostaglandin E2 (PGE2) and nitric oxide (NO) production in Raw 264.7 macrophages. In addition, GSK-J4 treatment significantly inhibited the generation of both PGE2 and NO after LPS stimulation in a dose-dependent manner as compared with the LPS only treated cells. However, the level of PGE2 and NO was not affected when Raw 264.7 cells were treated only with GSK-J4 (Figure 6). NSC-398 (a selective inhibitor of COX-2) and L-N6-(1-iminoethyl) lysine (_L_-NIL, a relatively selective inhibitor of iNOS) were used as a positive control.

### 2.6. The Inhibition of Jmjd3 Activity Alleviates LSS-Induced Mechanical Allodynia

**Figure 7:**
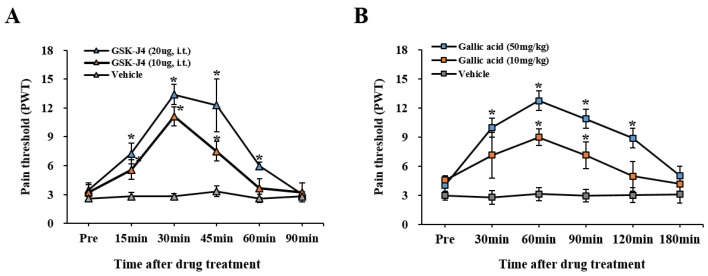
Blocking Jmjd3 activity alleviated LSS-induced mechanical allodynia. Thirty days after compression, rats underwent a pre-pain test (Pre) and were then injected with GSK-J4 (10 or 20 μg, i.t.) or Gallic acid (10, 50 mg/kg, i.p.). (**A**,**B**) Mechanical allodynia by von Frey filaments (n = 8). Data are presented as the mean ± SEM. * *p* < 0.05 vs. vehicle.

Finally, to determine the role of Jmjd3 in chronic mechanical allodynia, GSK-J4 (10 or 20 μg) was administered via i.t. into the cauda equina compression injured rats 30 d after LSS. At 30 min and 60 min after injection, GSK-J4 treatment exhibited significant increases in the mechanical pain threshold as compared to the vehicle control (at 30 min, 20 μg GSK-J4: 13.39 ± 1.05; 10 μg GSK-J4: 11.14 ± 0.84; Vehicle: 2.81 ± 0.31, *p* < 0.05) (Figure 7A). In addition, when a novel Jmjd3 inhibitor, gallic acid (10 or 50 mg/kg) [25], was treated via intraperitoneal (i.p.), the threshold of the mechanical PWT significantly increased from 30 min after injection as compared to vehicle-treated rats (at 60 min, 50 mg/kg Gallic acid: 12.78 ± 1.12; 10 mg/kg Gallic acid: 9.01 ± 0.87; Vehicle: 3.15 ± 0.68, *p* < 0.05) (Figure 7B). These results indicate that Jmjd3 mediates chronic neuropathic pain in LSS rats.

## 3. Discussion

Our present study demonstrates the role of histone H3K27 demethylase Jmjd3 in blood-nerve barrier hyperpermeability, macrophage infiltration, and neuroinflammation, which results in chronic neuropathic pain in the LSS rat model. Jmjd3 expression and activity were gradually increased in the cauda equina of chronic neuropathic pain developed rats until 1 month after a compression injury. Jmjd3 was induced in a blood vessel and infiltrated macrophages in injured the cauda equina, which increased blood-nerve barrier permeability by degrading tight junction proteins, such as ZO-1, occludin, and claudin-5, in endothelial cells and mediated neuroinflammation after the compression injury. In addition, the expression and activity of both MMP-2 and MMP-9 were also up-regulated in the cauda equina of LSS rats, and was significantly inhibited by a Jmjd3 inhibitor, GSK-J4, implying that Jmjd3 was involved in MMP-2 and MMP-9 expression and activation. Furthermore, GSK-J4 has an analgesic effect on LSS-induced chronic mechanical allodynia, suggesting that Jmjd3 may mediate LSS-induced chronic neuropathic pain. Notably, we also found that gallic acid, which has recently been reported to inhibit Jmjd3 activity, significantly alleviated LSS-induced mechanical allodynia.

LSS is attributed to the compression of cauda equina fibers and sensitization of the central and peripheral nervous systems. Nerve injury (at the peripheral and central nervous system) can lead to abnormal or overactive activity in regions of damaged nervous tissue, which is associated with severe and intractable neuropathic pain [26]. However, the therapy strategy for LSS pain is very limited because the understanding of the pathogenesis and molecular mechanism of this disease has not been uncovered very well. It has been reported that a robust neuroimmune response contributes to the development and maintenance of central/peripheral neuropathic pain. Indeed, after central/peripheral nerve injury, the soluble pro-inflammatory mediators and recruitment of immune cells to the site of nerve injury, the dorsal root ganglion (DRG), and the spinal cord [27,28,29] are increased. Recently, we showed that a significant amount of macrophages infiltrated into the cauda equina in the chronic stage after a compression injury and that COX-2, an inflammatory mediator, was expressed in macrophages [12]. In other neuropathic and inflammatory pain conditions, macrophages are also known to play important roles through phagocytosis, secretion of inflammatory mediators, and angiogenic processes by infiltrating into the damaged nerves and dorsal root ganglia [30,31]. This evidence suggests that macrophage infiltration control into the lesion site is an important factor in the development of neuropathic pain. In the present study, we showed that the breakdown of the blood-nerve barrier in an LSS-induced neuropathic pain model and blocking the hyperpermeability of the blood-nerve barrier inhibited the infiltration of macrophages into the cauda equina fibers (Figure 3). Dysregulation of the blood-nerve barrier is frequently associated with various types of peripheral neuropathies [32,33,34]. Several reports showed that blood-nerve barrier dysfunction contributes to the development of neuropathic pain after sciatic nerve ligation [35] or sciatic nerve chronic construction injury [36]. Although damage to the blood-nerve barrier has been previously reported in animal models of spinal stenosis [37,38], to the best of our knowledge, this is the first study to show the relationship between macrophage infiltration and the progression of neuropathic pain in a lumbar spinal stenosis animal model.

Previously, we demonstrated that histone K3K27 demethylase Jmjd3 is up-regulated in the injured blood vessel after a traumatic spinal cord injury and is involved in blood-spinal cord barrier disruption [19,25]. We also found that Jmjd3 is required for MMPs gene expressions, such as MMP-2, MMP-3, and MMP-9, in bEnd.3 endothelial cells after OGD/reperfusion injury [19], indicating that the regulation of Jmjd3 could be a new strategy to protect against the blood-brain barrier/blood-spinal cord barrier after injury. In this study, we found that a compression injury increased the expression and activity of Jmjd3 in microvascular endothelial cells of the cauda equina in LSS rats. Furthermore, blocking Jmjd3 activity inhibited blood-nerve barrier leakage, the expression and activation of MMP-2 and MMP-9, macrophage infiltration, and the expression of pro-inflammatory mediators such as TNF-α, IL-1β, COX-2, and iNOS after injury. Consequently, Jmjd3 inhibition ameliorated the development and/or maintenance of mechanical allodynia in LSS rats (Figure 7). Another important finding is that Jmjd3 was expressed in infiltrated macrophages and was involved in macrophage activation (see Figure 2 and Figure 6). It has been known that in macrophages, Jmjd3 expression is rapidly induced by pro-inflammatory stimuli including LPS through a nuclear factor-κB (NF-κB)-dependent mechanism, and Jmjd3 is recruited to the transcription start sites of over 70% of LPS-induced genes [23]. In this study, our data showed that PGE2 and nitric oxide were produced and secreted in the Raw 264.7 macrophages cell line when stimulated with LPS, whereas treatment with GSK-J4, a Jmjd3 inhibitor, significantly inhibited macrophage activation. In addition, GSK-J4 treatment significantly alleviated the expression and activation of MMP-2 and MMP-9 by LPS treatment (Figure 6). Taken together, our data suggest that Jmjd3 is involved in neuropathic pain development and/or maintenance after cauda equina compression, in part by inhibiting blood-nerve barrier damage and macrophage activation through regulating MMP-2 and MMP-9 expression.

It has been known that GSK-J4 inhibits Ubiquitously transcribed tetratricopeptide repeat on chromosome X (UTX)/Lysine Demethylase 6A (KDM6A) as well as Jmjd3. Although very little is known about the function of UTX in the nervous system, it was recently reported that UTX deletion improves recovery by triggering vascular regeneration and intrinsic neural regeneration after spinal cord injury [39,40]. However, in the present study, we focused on demonstrating the effect of Jmjd3 on blood-nerve barrier permeability and macrophage activity in the LSS animal model based on our previous study that Jmjd3 mediates MMP-2 and MMP-9 gene expression in endothelial cells [19]. Therefore, we think that there is no problem in interpreting the data on the investigation of the role of Jmjd3 in blood-nerve barrier damage followed by macrophage infiltration and macrophage activation in the LSS animal model. However, we cannot rule out the possibility that GSK-J4 affects UTX, which may be involved in the blood-nerve barrier dysfunction and macrophage activation in the LSS animal model.

Additionally, our recent report showed that gallic acid, a natural phenolic compound, exhibited a neuroprotective effect by inhibiting the expression and activation of Jmjd3, thereby preventing blood-spinal cord barrier disruption via down-regulating Jmjd3-mediated activation of MMP-3 and -9 after spinal cord injury [25]. In this study, when the cauda equina compression injured rats were treated with GSK-J4 as well as gallic acid as another Jmjd3 inhibitor, mechanical allodynia was significantly alleviated (Figure 7B). Our data suggest that targeting for Jmjd3 may provide an effective therapeutic strategy to relieve chronic neuropathic pain in LSS patients. 

## 4. Materials and Methods

### 4.1. Animals and Ethics Statement

Sprague-Dawley male rats (250–270 g; Samtako, Osan, Korea) were used in this study. The animals were maintained under a constant temperature (23 ± 1 °C) and humidity (60 ± 10%) under a 12 h light/dark cycle (light on 07:30–19:30 h) with ad libitum access to drinking water and food. The rats were housed one per cage (410 mm × 282 mm × 153 mm, transparent polycarbonate) with aspen shaving bedding and were fed a commercial diet (5L79, PMI Nutrition International, St Louis, MO, USA) and commercial standard chow (Lab Diet 5L79l Purina Mills, Richmond, IN, USA). All animal experiments were performed in accordance with the Guidelines of Animal Care Committee of the Kyung Hee University (permission number: KHSASP-19-096) and followed the Ethical issues of the International Association for the Study of Pain [41].

### 4.2. Cauda Equina Compression Injury

Cauda equina compression was induced based on our previous report [12]. Briefly, rats were anesthetized with chloral hydrate (500 mg/kg) by i.p. injection, and the back regions were shaved to expose the vertebral plate at the L4-S2 level. The ligamentum flavum between L4 and L5 was removed. Subsequently, a trapezoid-shaped silicon block (1.00 mm Length × 1.3~1.2 mm Width × 1.0 mm Height) was inserted into the epidural space under the L5 and L6 vertebral plate without disrupting the dural sac. For the sham-operated group, animals were only posteriorly open and drilled, without inserting a silicon block. Throughout the surgical procedure, body temperature was maintained at 37 ± 0.5 °C with a heating pad (Biomed S.L., Alicante, Spain). After the injury, muscles and skin were closed in layers, and the rats were placed in a temperature and humidity-controlled chamber overnight. Postoperatively, the rats received subcutaneously supplemental fluids (5 mL, lactated ringer) and antibiotics (gentamicin, 5 mg/kg, intramuscular injection) once daily for 5 d after surgery. Rats were housed one per cage with water and food easily accessible. Body weights and the remaining chow and water weight were recorded each morning for all animals.

### 4.3. Pain Behavioral Tests

Pain behavioral tests were performed as previously described [42]. Mechanical allodynia was assessed by PWT in response to probing with a series of calibrated von Frey filaments (3.92, 5.88, 9.80, 19.60, 39.20, 58.80, 78.40, and 147.00 mN, Stoelting, Wood Dale, IL; equivalent in grams to 0.4, 0.6, 1.0, 2.0, 4.0, 6.0, 8.0, and 15.0). The 50% withdrawal threshold was determined by using the up-down method [43]. In brief, rats were placed under transparent plastic boxes (28 cm × 10 cm × 10 cm) on a metal mesh floor (3 mm × 3 mm mesh). They were then left alone for at least 20 min of acclimation before sensory testing began. Testing was initiated with the filament whose bending force was 19.60 mN, in the middle of the series. The von Frey filament was applied to the plantar surface of each hind paw, and the most sensitive spot was determined by probing various areas with the 19.60 mN filament. In the absence of a hind paw withdrawal response to this filament, this process of searching for the sensitive spot was repeated with the next stiffer filament. Once the sensitive spot, by which a positive response had been elicited, was determined, this spot was touched with one of a series of eight von Frey filaments in an up-down fashion, in the following manner; a withdrawal response was cause to present the next weaker stimulus, and the lack of withdrawal led to the presentation of the next stronger stimulus. Stimuli were applied for 3–4 s to each hind paw while the filament was bent and presented at intervals of several seconds. A brisk hind paw withdrawal to the von Frey filament application was regarded as a positive response. Each filament was applied twice. PWT for each animal was averaged on both paw values, and subsequently, the values of the individual animals were averaged to obtain the group mean. All pain behavioral testing was performed by trained investigators who were blind as to the experimental conditions.

### 4.4. Drug Administration

At 30 d after cauda equina compression, we selected only those rats that weighed between 350 and 380 g and developed with mechanical allodynia (PWT; 2.5–4.0 g), and then divided them randomly into each experimental group. More than 85% of rats met this criterion. A Jmjd3 inhibitor, GSKJ4 (10 or 20 µg; Merck, Darmstadt, Germany) was dissolved in 50% dimethyl sulfoxide (DMSO) in saline and administered intrathecally with 10 μL as described [42]. Vehicle groups received equivolumetric injections of 50% DMSO. For some experiments, gallic acid (Cayman Chemical, Ann Arvor, MI, USA) was dissolved in saline and injected via i.p. at a dose of 50 mg/kg or 100 mg/kg. Vehicle control rats received equivolumetric i.p. injections of saline at the corresponding time points. For the sham-operated control, animals were exposed to the spine without the insert of a silicon block and did not receive any pharmacological treatment.

### 4.5. Tissue Preparation

Thirty minutes after GSKJ4 injection (at the time of peak effect), the animals were anesthetized with chloral hydrate (500 mg/kg) and perfused transcardially with 0.1M PBS (pH 7.4) followed by 4% paraformaldehyde in PBS. The cauda equina segments were embedded in the OCT compound and then cut at 10 μm thickness on a cryostat (CM1850; Leica, Wetzlar, Germany). For molecular work, rats were perfused at 30 min after GSK-J4 treatment with 0.1 M PBS and a compressed cauda equina segment (1 cm) was isolated and frozen at −80 °C until use as previously described [19].

### 4.6. Immunohistochemistry and Immunocytochemistry

Frozen sections of cauda equine or fixed bEnd.3 cells were processed for immunostaining with antibodies against Jmjd3 (1:100, Abcam, Cambridge, MA, USA), ED-1 (CD68, 1:200; Serotec, Raleigh, NC, USA), RECA1 (1:100; Serotec, Raleigh, NC, USA), and ZO-1 (1:300, Invitrogen, Carlsbad, CA, USA) as previously described [19]. For double labeling, FITC or cy3-conjugated secondary antibodies (Jackson ImmunoResearch, West Grove, PA, USA) were used. Additionally, nuclei were labeled with DAPI according to the protocol of the manufacturer (Molecular Probes, Eugene, OR, USA). In all controls, reaction to the substrate was absent if the primary antibody was omitted or if the primary antibody was replaced by a non-immune, control antibody. Serial sections were also stained for histological analysis with Cresyl violet acetate. Fluorescence labeled signal was detected by a fluorescence microscope (BX51, Olympus, Japan), and capture of images and measurement of signal colocalization was determined by MetaMorph software (Molecular Devices, Sunnyvale, CA, USA). To count the number of macrophages, eight sections were selected at 1-mm intervals in the compressed region of the cauda equina, and the total number of ED-1-positive cells per section was averaged.

### 4.7. Western Blot

Total protein from the cauda equina segments including the compression site was prepared and Western blot analysis was performed as previously described [12]. The primary antibodies used in Western blot are as follows; Jmjd3 (1:1000, Abcam), H3K27Me3 (1:1000, Abcam), ZO-1 (1:1000, Invitrogen), occludin (1:1000, Invitrogen), Claudin-5 (1:1000, Invitrogen), and ED-1 (1:1000, Serotec). As a loading control, β-tubulin (1:10,000; Sigma, St. Louis, MO, USA) was used. Quantification of bands was performed by AlphaImager software (Alpha Innotech Corporation, San Leandro, CA, USA).

### 4.8. Gelatin Zymography

At 30 d after the compression injury, total protein (50 µg) was loaded on a Novex 10% zymogram gel (EC61752; Invitrogen) and separated by electrophoresis with 100 V (19 mA) at 4 °C for 6 h. The gel was then incubated with renaturing buffer (2.5% Triton X-100) at room temperature for 30 min to restore the gelatinolytic activity of the proteins. After incubation with developing buffer (50 mM Tris-HCl, pH 8.5, 0.2 M NaCl, 5 mM CaCl_2_, 0.02% Brii35) at 37 °C for 24 h, the gel was stained with 0.5% Coomassie blue for 24 h and then destained with 40% methanol containing 10% acetic acid until the appropriate color contrast was achieved. Clear bands on the zymogram were indicative of gelatinase activity. The relative intensity of zymography (relative to sham or vehicle) was measured and analyzed by AlphaImager software (Alpha Innotech Co., San Leandro, CA, USA). The background was subtracted from the optical density measurements. Experiments were repeated three times and the values obtained for the relative intensity were subjected to statistical analysis.

### 4.9. RNA Isolation and RT-PCR

Total RNA from the cauda equina segments including compression site was prepared and RT-PCR for TNF-α, IL-1β, IL-6, COX-2, iNOS, MMP-2, MMP-9 and GAPDH were performed as previously described [42]. The sequences of the primers are as follows (5′-3′): TNF-α forward, 5′-CCC AGA CCC TCA CAC TCA GAT-3′; reverse, 5′-TTG TCC CTT GAA GAG AAC CTG-3′; IL-1β forward, 5′-GCA GCT ACC TAT GTC TTG CCC GTG-3′, reverse, 5′-GTC GTT GCT TGT CTC TCC TTG TA-3′; IL-6 forward, 5′-AAG TTT CTC TCC GCA AGA TAC TTC CAG CCA-3′; reverse, 5′-AGG CAA ATT TCC TGG TTA TAT CCA GTT-3′; COX-2 forward, 5′-CCA TGT CAA AAC CGT GGT GAA TG-3′; reverse, 5′-ATG GGA GTT GGG CAG TCA TCA G-3′; iNOS forward, 5′-CTC CAT GAC TCT CAG CAC AGA G-3′, reverse, 5′-GCA CCG AAG ATA TCC TCA TGA T-3; MMP-2 forward, 5′-ACC ATC GCC CAT CAT CAA GT-3′, reverse, 5′-CGA GCA AAA GCA TCA TCC AC-3′; MMP-9 forward, 5′-AAA GGT CGC TCG GAT GGT TA-3′, reverse, 5′-AGG ATT GTC TAC TGG AGT CGA-3′. GAPDH forward, 5′-AAC TTT GGC ATT GTG GAA GG-3′; and reverse, 5′-GGA GAC AAC CTG GTC CTC AG-3′. 

### 4.10. Endothelial Cell Culture and OGD/Reperfusion

Mouse brain microvessel endothelial cell line, bEnd.3, was cultured and subjected to OGD/reperfusion injury using a humidified anaerobic chamber (APM-30D, Astec, Fukuoka, Japan) as previously described [21]. In brief, bEnd.3 cells were cultured with Dulbecco modified Eagle medium (DMEM, Sigma) containing 10% FBS to confluence; the culture medium was then replaced with glucose-free DMEM (Sigma) and then placed in a humidified anaerobic chamber (0.1% O_2_, 5% CO_2_, and 94.9% N_2_). After 6 h of OGD, cells were further cultured in DMEM with 25 mM glucose under normoxia (reoxygenation). Control cells were cultured in DMEM with 25 mM glucose under normoxia. GSK-J4 (4 μM) was dissolved in 0.1% dimethylsulfoxide (DMSO) and treated for 30 min before OGD. For control, 0.1% DMSO was treated.

### 4.11. Measurement of Transendothelial Electrical Resistance (TEER)

Endothelial permeability was determined by measuring TEER. TEER across the monolayers grown on the filter membranes was measured using the Millicell ERS Voltohmmeter (Millipore, Burlington, MA, USA), and the values are shown as Ω·cm^2^ based on culture inserts. The TEER of cell-free inserts was subtracted from that of filters with cells, as previously described [19].

### 4.12. RAW 264.7 Macrophages Culture

RAW 264.7, a murine macrophage cell line (American Type Culture Collection, Rockville, MD, USA) was grown in DMEM containing 10% fetal bovine serum and antibiotics (100 U/mL penicillin and 100 μg/mL streptomycin) in a humidified atmosphere at 37 °C with 5% CO_2_ as described [44]. The cells were seeded (1 × 10^6^ cells/well) in a 6-well plate. The next day, cells were treated with GSKJ4 (3, 10, or 30 μM, final 0.01% DMSO in saline) for 30 min and then stimulated with LPS (1 μg/mL) for 24 h. _L_-NIL hydrochloride (40 μM, R&D systems) and NS398 (10 nM, Tocris Bioscience, Bristol, UK) were used as NO and PGE2 inhibitors, respectively.

### 4.13. Determination of NO and PGE2 Production

RAW 264.7 macrophage cells were plated at 2 × 10^5^ cells per well in 24-well plates and then incubated with or without LPS (1 μg/mL) in the absence or presence of GSK-J4 for 24 h. Nitrite levels in the culture media were determined using the Griess reaction assay and presumed to reflect NO levels. The optical density was measured at 540 nm by a microplate reader. For the PGE2 assay, dilutions of the cell culture medium were assayed for PGE_2_. PGE_2_ levels in the cell culture medium were determined using a colorimetric competitive enzyme-linked immunosorbent assay (ELISA) kit (Enzo Life Science, NY, USA) according to the manufacturer’s instructions.

### 4.14. Measurement of Blood-Nerve Barrier Permeability

At 30 d after compression injury, the blood-nerve barrier permeability was measured with Evans blue dye as previously described [21] with minor modifications. Immediately after GSK-J4 or saline was administered, 2% of Evans blue was dissolved in saline and injected via i.p. into rats (5 mL). After 3 h, the rats were perfused with PBS and the compressed cauda equina tissues (1 cm) were removed and homogenized in 50% trichloroacetic acid solution. The homogenates were centrifuged at 10,000× *g* for 10 min, then the supernatants were collected. The amount of Evans blue dye in the cauda equina was measured using a spectrophotometer at an excitation wavelength of 620 nm and an emission wavelength of 680 nm. Dye in samples was determined as micrograms per gram of tissue from a standard curve plotted using known amounts of dye.

### 4.15. Statistical Analysis

Data are presented as mean ± SD or SEM. Comparison between experimental groups was evaluated for statistical significance using the unpaired Student’s t-test. Multiple comparisons between groups were performed by one-way ANOVA. Some behavioral scores were analyzed by repeated ANOVA measures. Dunnett’s case comparison was used as post hoc analysis. The size of groups was expressed by the number of animals in each group. Statistical significance was accepted with *p* < 0.05. All statistical analyses were performed by using SPSS 15.0 (SPSS Science, Chicago, IL, USA).

## 5. Conclusions

Overall, our results show that Jmjd3, expressed in the blood vessels and infiltrated macrophages in the cauda equina, likely plays a pivotal role in blood-nerve barrier permeability and chronic neuropathic pain after cauda equina compression. This suggests that the inhibition of Jmjd3 can be considered as an important therapeutic strategy to alleviate chronic pain by blocking blood-nerve barrier hyperpermeability and neuroinflammation in LSS patients.

## Data Availability

The data that support the findings of the study are available from the corresponding authors upon reasonable request.
